# Diagnostic Journey to Granulomatosis with Polyangiitis with Rare Hepatic and Suspected Splenic Involvement: A Case Report

**DOI:** 10.3390/diseases14090328

**Published:** 2026-09-09

**Authors:** Aleksandra Dasic, Dragan Vasin, Maja Stojanovic, Dragana Jovanovic, Anja Kisic, Ana Marija Tomic, Rada Miskovic

**Affiliations:** 1 Clinic of Allergy and Immunology, University Clinical Center of Serbia, 11000 Belgrade, Serbia; 2 Center for Radiology, Department of Emergency Radiology, University Clinical Center of Serbia, 11000 Belgrade, Serbia; 3 Faculty of Medicine, University of Belgrade, 11000 Belgrade, Serbia; 4 Institute of Pathology, Faculty of Medicine, University of Belgrade, 11000 Belgrade, Serbia

**Keywords:** granulomatosis with polyangiitis, hepatic granuloma, splenic involvement, sarcoidosis, case report

## Abstract

Sarcoidosis and granulomatosis with polyangiitis (GPA) are the most common non-infectious causes of granulomatous lung disease. However, they can sometimes be difficult to distinguish due to overlapping clinical, radiological, and histopathological features. A 54-year-old man was considered to have sarcoidosis in 2018. Treatment with glucocorticoids led to improvement, but the patient self-discontinued it in 2021. In January 2022, he was readmitted due to recurrent cough and fatigue, who then developed purpuric lesions on the lower extremities, renal impairment, and abnormal results for liver function tests. Chest computed tomography showed lymphadenopathy, consolidations, and nodular lesions. Liver and spleen lesions were detected on abdominal ultrasound, and liver biopsy was performed revealing necrotizing granulomas. Strongly positive proteinase 3 (PR3) anti-neutrophil cytoplasmic antibody (ANCA) together with clinical, radiological, and histopathological findings supported the diagnosis of GPA. Treatment with methylprednisolone and cyclophosphamide led to clinical and partial laboratory improvement. Unfortunately, the patient died suddenly, reportedly in association with massive hemoptysis. Ongoing reassessment during the disease course is essential for accurate diagnosis and optimal management. This case highlights the diagnostic challenges posed by overlapping features of sarcoidosis and GPA, and the rare presence of hepatic necrotizing granulomas with concurrent radiologically suspected granulomatous splenic involvement. The limited data reported in the literature make this case particularly unique and diagnostically significant.

## 1. Introduction

The differential diagnosis of granulomatous lung disease presents considerable challenges due to the overlapping clinical, radiological, and histopathological features across various entities. The most common causes among non-infectious etiologies are sarcoidosis and granulomatosis with polyangiitis (GPA) [1]. Although both diseases are fundamentally characterized by granulomatous inflammation, their underlying immunopathogenic mechanisms are different: GPA is primarily neutrophil-mediated whereas sarcoidosis is a macrophage- and T-cell-driven disease [2,3]. This is also reflected in the distinct histological features of granulomas: necrotizing in GPA and non-necrotizing in sarcoidosis [1]. Distinguishing between these two entities is particularly challenging in early stages, when diagnostic criteria may not be fully met.

Granulomatosis with polyangiitis is a necrotizing AAV that predominantly affects small to medium blood vessels in the upper and lower respiratory tract and kidneys. However, its broad spectrum of clinical presentation and variable organ involvement may delay recognition and make timely diagnosis challenging [2].

In this paper, we describe a rare case of a patient initially considered to have pulmonary sarcoidosis, who subsequently presented with clinical, radiological, and immunoserological findings consistent with GPA, accompanied by unusual granulomatous involvement of the liver and radiologically suspected granulomatous involvement of the spleen. After a thorough review of the literature, we identified only a limited number of reports describing hepatic granulomas and/or radiologically suspected granulomatous splenic involvement in GPA, highlighting the rarity of this presentation and associated diagnostic challenges.

This case underscores the diagnostic complexity of granulomatous diseases and emphasizes the importance of longitudinal clinical, radiological, and pathological evaluation in establishing the accurate diagnosis. The CARE checklist is provided in Supplementary Materials.

## 2. Case Presentation

A 54-year-old male non-smoker presented in 2018 with fatigue, dry cough, arthralgia, erythematous rash on the lower limbs, and subcutaneous forearm nodules. Chest CT revealed micronodular lesions in the lung parenchyma, along with retroperitoneal and mediastinal lymphadenopathy (Figure A1). Although flexible bronchoscopy with lymph node biopsy yielded nonspecific histopathological findings, he was considered to have sarcoidosis at an outside clinic. Initial treatment with oral glucocorticoids (GC), prednisone 20 mg/day, followed by tapering, led to symptomatic improvement. Follow-up was interrupted due to the COVID-19 pandemic. During this period, the patient remained unvaccinated against COVID-19 and had no documented SARS-CoV-2 infection. He self-discontinued GC therapy in 2021 for unknown reasons.

In January 2022, he was admitted to the pulmonology clinic due to malaise and productive cough with thick sputum, symptoms that had been present for one month. Laboratory findings included leukocytosis, thrombocytosis, normocytic anemia, and elevated inflammatory markers (erythrocyte sedimentation rate of 70 mm/h, C-reactive protein of 203 mg/L). Renal impairment was evident (creatinine of 150 umol/L, estimated glomerular filtration rate of 42 mL/min/1.73 m^2^). Urinalysis demonstrated presence of dysmorphic erythrocytes, sterile leukocyturia, and proteinuria (1.53 g/24 h), but the patient was not motivated for the kidney biopsy. Laboratory findings are presented in detail in Table 1.

A repeated chest CT showed persistent mediastinal and hilar lymphadenopathy, parahilar consolidations, and diffuse irregular nodular lesions with partial cavitation (Figure A1). Bronchoscopy revealed edematous, hyperemic mucosa with white nodules. Nevertheless, the histopathology of mucosal and lymph node biopsies remained nonspecific, likely due to sampling limitations. The clinical course was further complicated by the development of fever, oral ulcerations, and purpuric vasculitic lesions on the lower extremities (Figure A2). Immunological workup performed in March 2022 demonstrated strongly positive cytoplasmic anti-neutrophil cytoplasmic antibodies (ANCAs) at a titer of 1:320 (indirect immunofluorescence), with elevated proteinase 3-ANCA (PR3-ANCA > 200 RU/mL, ELISA). A diagnosis of GPA was made. Laboratory markers showed elevated chitotriosidase activity, while serum angiotensin-converting enzyme (ACE) levels remained within normal limits on repeated measurements. Ultrasonography identified hypovascular focal lesions in the liver and spleen, prompting a targeted percutaneous core liver biopsy. Histopathological analysis revealed large necrotizing granulomas located within the portal tracts, composed predominantly of epithelioid histiocytes surrounding central zones of necrosis and fibrosis (Figure A3). Special stains, including Ziehl–Neelsen and PAS, were negative for microorganisms. Comprehensive microbiological investigations to exclude infectious etiology of these granulomas were performed and yielded negative results (Table 2).

The patient was initially treated with methylprednisolone at a dose of 1.5 mg/kg according to body weight, followed by oral prednisone at 0.75 mg/kg based on body weight with subsequent tapering. Rituximab was considered for induction therapy but was not immediately available due to administrative limitations in our country. Cyclophosphamide was administered as intravenous pulse therapy at a dose of 1000 mg every 4 weeks according to the modified NIH protocol used in our country for severe GPA. This locally used monthly regimen differs from the contemporary recommended by guidelines for cyclophosphamide pulse regimens for AAV. Our patient received a total of two pulses, corresponding to a cumulative cyclophosphamide dose of 2000 mg. During the second cycle of cyclophosphamide pulse, worsening proteinuria was observed. In the absence of other clinical and laboratory signs of worsening GPA activity (urea and creatinine levels and eGFR were improving), and given the suspicion of a urinary tract infection, two additional intravenous methylprednisolone pulses of 1000 mg each were administered alongside empiric antibiotic therapy. Pneumocystis jirovecii prophylaxis with trimethoprim–sulfamethoxazole (TMP–SMX of 160/800 mg) was administered three times weekly. Repeated chest radiography showed no evidence of disease progression, and a follow-up CT scan was scheduled after the third treatment cycle.

Although the patient initially showed partial clinical response to glucocorticoid and cyclophosphamide therapy, he died suddenly at home, reportedly in association with massive hemoptysis. The family declined consent for a post-mortem examination; therefore, the exact cause and mechanism of death could not be established.

The clinical course of the patient is illustrated in the timeline (Figure A4).

## 3. Discussion

Sarcoidosis and GPA are both systemic immune-mediated granulomatous diseases of unknown cause. Clinical presentation and radiological findings of these two entities may overlap; therefore, the diagnosis of GPA requires integrated assessment including ANCA testing and histological verification, when available [1].

In GPA, the dominant pathogenic mechanism is neutrophil-mediated. ANCA binding to PR3 expressed on primed neutrophils leads to neutrophil degranulation, reactive oxygen species production, and formation of neutrophil extracellular traps (NETs), culminating in endothelial damage and amplification of the inflammatory cascade. Dendritic cells, activated by various components of NETs, including PR3, further induce the activation of T and B lymphocytes, thereby initiating the autoimmune response and ANCA production [2].

In sarcoidosis, macrophages are considered the primary effector cells. When activated, they secrete IL-6, IL-12, IL-18, and TGF-β, promoting the differentiation of naïve CD4+ T cells into Th1 and Th17 subpopulations, while simultaneously suppressing regulatory T-cell-mediated anti-inflammatory mechanisms. Differentiation toward the Th1 phenotype is primarily responsible for the acute granulomatous inflammation, while the dominant mechanism in chronic disease remains insufficiently understood. B cells have been detected within granulomas, although their precise function in the pathogenesis remains unclear [3,4].

Emerging evidence suggests that certain aspects of the immunopathogenic mechanisms of sarcoidosis and AAV may share common features, such as the activation of Th1 and Th17 immune responses and dysfunction of regulatory T cells. B-cell involvement is well established in GPA through its role in autoantibody production but is still not fully understood in sarcoidosis [3]. The potential role of B cells in the pathogenesis of sarcoidosis is evidenced by the clinical use of rituximab in these patients, as reported in the literature. This suggests that certain immunopathogenic mechanisms may overlap between these two diseases [4].

The absence of standardized diagnostic criteria for sarcoidosis poses a significant challenge. Even though sarcoidosis is defined by the presence of non-necrotizing granulomas in affected organs, current diagnostic protocols depend on the integration of compatible clinical manifestations, histopathological evidence, and exclusion of alternative granulomatous diseases. Due to the lack of universally accepted criteria, the diagnosis of sarcoidosis remains less definitive compared to conditions with established diagnostic standards [5,6]. Since our patient was initially considered to have sarcoidosis at an outside institution, the results of the initial laboratory investigations were unavailable for review. Furthermore, the differential diagnoses considered during the initial assessment and the rationale for not performing immunological testing could not be determined. In retrospect, cavitating pulmonary nodules were already present on the lung CT performed in 2018 (Figure A1b), although they were neither described nor recognized as such at that time. Although these findings are not pathognomonic for GPA, their presence should have prompted consideration of GPA and ANCA testing at that time. These radiological findings may have represented an early manifestation of GPA in our patient, given that cavitating pulmonary lesions are atypical for sarcoidosis.

Elevated chitotriosidase levels in serum and bronchoalveolar lavage are a recognized feature of sarcoidosis. Although not specific for diagnosis, they serve as a valuable prognostic marker and reflect therapeutic response to glucocorticoids. Chitotriosidase may also be considered a relevant biomarker of inflammation in other respiratory diseases characterized by chronic inflammation and macrophage activation [7]. However, in the context of GPA, while serum chitotriosidase levels have been reported to be relatively elevated compared to healthy controls, they do not show a significant correlation with disease activity, in contrast to findings observed in sarcoidosis [8].

A wide variety of entities have clinical and/or radiological features similar to sarcoidosis and are therefore called sarcoidosis mimics. These include mycobacterial and fungal infections, pneumoconiosis, malignancies, localized reactions to foreign bodies or drugs, necrotizing sarcoid granulomatosis, and ANCA-associated vasculitis (AAV). Extrapulmonary manifestations usually provide important clues in the differential diagnosis, for example upper airway and/or kidney involvement in GPA [9].

Significant eosinophilia was initially observed in our patient (Table 1), raising the suspicion of a eosinophilic granulomatosis with polyangiitis (EGPA) as the differential diagnosis. Using the 2022 American College of Rheumatology/European Alliance of Associations for Rheumatology (ACR/EULAR) classification criteria for EGPA, our patient had eosinophilia (+5 points), positive PR3-ANCA (−3 points), and hematuria (−1 point), resulting in a total score of one point, which is insufficient for EGPA classification. In addition, the patient had no clinical features suggestive of obstructive airway disease, nasal polyps, and/or mononeuritis multiplex. Myeloperoxidase (MPO)-ANCAs were tested on multiple occasions and were consistently negative. Due to the observed eosinophilia, microbiological evaluation included an extensive parasitological workup (Table 2).

In a case of a febrile patient with hepatic and splenic granulomas, granulomatous lesions of unknown significance (GLUS syndrome) should be considered in the differential diagnosis. However, our patient had normal serum ACE levels and no B cells in histopathological examination of the hepatic granuloma, findings that argue against this entity [9].

Within the spectrum of granulomatous diseases, necrotizing sarcoid granulomatosis (NSG) warrants consideration. Radiologically presenting as nodular masses, an NSG diagnosis relies on histopathology showing sarcoid-like granulomas with extensive necrosis and diffuse vasculitis. While some authors classify NSG as a variant of sarcoidosis, its frequent misdiagnosis as tuberculosis highlights the importance of accurate differentiation and thorough exclusion of other causes of granulomatous inflammation [10,11].

Liver and spleen involvement are common extrapulmonary manifestations of sarcoidosis, alongside skin lesions and uveitis [12]. Hepatic involvement in sarcoidosis is described in almost one-third of patients and presents mostly as elevated liver function tests (LFTs) [13]. The most commonly observed radiological findings in these patients are hepatomegaly and/or hypodense nodules localized in the portal and periportal areas. Splenic involvement is described in 15% of patients with sarcoidosis, usually as splenomegaly or splenic nodules that tend to coalesce [12].

Incidence of liver involvement in patients with GPA is only 5–11%, according to the literature [14]. It is usually asymptomatic and presents as elevated LFT, showing both cholestatic and hepatocellular patterns [9,13,15,16]. Apart from hepatomegaly, nodular changes have also been described, but only in several case reports [14,17]. Histopathologically, GPA is typically characterized by necrotizing granulomatous inflammation, palisading histiocytes and small-vessel vasculitis. However, histological evidence of vasculitis demonstrated on liver biopsy is extremely rare [16] and in some patients extravascular granulomatous inflammation may be the dominant feature [18], as was the case in our patient. Hepatic involvement was associated with the higher disease activity and indicated poorer outcome in patients with GPA [19].

Frequency of spleen involvement in patients with GPA is yet unknown. It typically presents as homogeneous splenomegaly and/or hypodense, irregular nodular lesions that often tend to coalesce and regress successfully with therapy [20]. In contrast, the splenic lesions observed on ultrasound in our patient were hypovascular, focal, and regular in shape, and were considered compatible with granulomatous involvement based on the similar histopathologically proven granulomatous lesions identified in the liver. Extremely rarely, splenic involvement in GPA can present with spontaneous splenic rupture due to splenic infarction [21]. Histopathological confirmation of splenic involvement is rarely obtained, as splenic biopsy in patients with vasculitic changes carries a significant risk of bleeding.

In our patient, despite the absence of the splenic biopsy, lesions in the spleen were suspected to represent granulomatous involvement, based on their similar ultrasonographic appearance to the lesions observed in the liver. To the best of our knowledge, despite an extensive literature review, no previously reported cases of suspected splenic granulomatous involvement in the context of GPA have been identified.

Nonspecific and rare localization of granulomas in GPA can lead to diagnostic uncertainty or delay in more than 30% of patients. In particular, the detection of hepatic granulomas accompanied by focal splenic lesions, as seen in our case, is very uncommon and may mimic neoplastic or infectious processes, underscoring the diagnostic challenges in such cases [22]. Atypical localization makes timely diagnosis difficult and requires histopathological verification whenever possible.

Since autopsy was not performed in our patient, the exact cause of death remains unknown. Potential mechanisms that should be considered include diffuse alveolar hemorrhage, erosion of adjacent blood vessels by cavitary lesions, superimposed pulmonary infection in the setting of applied intensive immunosuppressive therapy, and pulmonary thromboembolism. Rupture of a pulmonary artery pseudoaneurysm and carcinoma are less likely mechanisms, as no such abnormalities were identified on available radiological imaging.

## 4. Conclusions

Distinguishing GPA from sarcoidosis can be particularly challenging, as the two entities often exhibit overlapping clinical and radiological features, especially in the early stages. This case underscores the potential for GPA to closely mimic sarcoidosis at presentation, emphasizing the diagnostic complexity, while highlighting the critical importance of a thorough differential diagnosis and ongoing longitudinal assessment. Histopathological confirmation in cases with atypical organ involvement is essential to ensure diagnosis confirmation and guide appropriate management.

## Figures and Tables

**Table 1:** Relevant laboratory and immunoserological analysis during the disease. GCS—glucocorticosteroids; CYC—cyclophosphamide; IIF—indirect immunofluorescence; ELISA—enzyme-linked immunosorbent assay); anti-GBM—anti-glomerular basement membrane.

Laboratory Tests (Normal Range)	January 2022 (Before GCS)	March 2022 (During GCS)	April 2022 (Before CYC)	June 2022 (During CYC Therapy)
**White blood cells** (3.4–9.7 × 10^9^/L)	16.2	10.5	17.1	12.6
**Neutrophils** (2.1–6.5 × 10^9^/L)	11.8	7.5	15.8	8.7
**Lymphocytes** (1.2–3.4 × 10^9^/L)	2.5	1.1	0.5	2.6
**Eosinophils** (0.0–0.4 × 10^9^/L)	0.9	1.4	0	0.2
**Red blood cells** (4.34–5.72 × 10^12^/L)	4.83	3.79	4.0	4.2
**Hemoglobin** (138–175 g/L)	128	90	107	106
**Platelets** (158–424 × 10^9^/L)	589	753	185	415
**Erythrocyte** **sedimentation rate** (<10 mm/h)	70	100	60	38
**C-reactive protein** (0–5 mg/L)	203	136.6	142.1	35.1
**Fibrinogen** (2–4 g/L)	4.3	3.6	2.4	5.1
**Ferritin** (30–400 ug/L)	1303.8	1624.0	1295.1	655.8
**Creatinine** (59–104 umol/L)	150	246	159	144
**Urea** (2.5–7.5 mmol/L)	7.6	10.9	14.8	9.5
**eGFR** (mL/min/1.73 m^2^)	42	24	40	44
**Urine sediment**	dysmorphic erythrocytes, proteinuria, sterile leukocyturia	dysmorphic erythrocytes, proteinuria, sterile leukocyturia	erythrocytesproteinuria	proteinuria, leukocyturia
**24 h urinary proteinuria** (<0.15 g/24 h)	1.53	0.725	0.78	1.43
**Alanine** **aminotransferase** (0–41 U/L)	27	52	11	20
**Aspartate** **aminotransferase** (0–37 U/L)	27	53	25	13
**Gamma-glutamyl transferase** (0–55 U/L)	98	141	87	53
**Alkaline** **phosphatase** (40–120 U/L)	120	165	99	90
**Lactate dehydrogenase** (220–460 U/L)	615	673	240	269
**Angiotensin-converting enzyme** (12–68 U/L)	-	23	18	-
**Chitotriosidase** (1.8–146 nmol/mL/h)	173.3	275.9	131.0	-
**24 h urinary calcium** (<7.5 mmol/L)	-	1.29	-	-
**Rheumatoid Factor** (0–30 IU/mL)	-	146	-	<20
**Immunoglobulin E** (0–100 IU/mL)	-	502	-	-
**Immunoglobulin G** (5.4–18.2 g/L)	-	15.0	-	14.2
**ANA HEp2, IIF**	-	negative	-	-
**ANCA, IIF**	-	cANCA 1:320	-	-
**Anti-PR3** (<20 RU/mL), ELISA	-	>200	-	50
**Anti-MPO** (<20 RU/mL), ELISA	-	0.01	-	0.01
Complement component 3 (0.82–1.85 g/L)	-	1.26	1.33	-
Complement component 4 (0.12–0.36 g/L)	-	0.18	0.25	-
Anti-GBM	-	negative	negative	-

**Table 2:** Relevant microbiological analysis performed.

Microbiological Tests			
**Nasopharyngeal swab**	**bacteria and fungi**		Negative
	**PCR panel**	***Influenza A***	Negative
		***Influenza B***	Negative
		***Respiratory Syncytial Virus***	Negative
**Sputum analysis**	**Ag test for ** ***SARS-CoV-2***		Negative
	**bacteria and fungi**		Negative
	***Mycobacterium tuberculosis***	Ziehl–Neelsen stain	Negative
		MGIT	Negative
		Lowenstein	Negative
**Cultures of bronchial aspirate**	***Aspergillus *** **galactomannan**		Negative
	***Candida *** **mannan**		Negative
	***Mycobacterium tuberculosis***	Ziehl–Neelsen stain	Negative
		MGIT	Negative
		Lowenstein	Negative
**Serological testing**	**viruses**	***Human Immunodeficiency *** ***Virus***	Negative
		***Hepatitis B Virus***	Negative
		***Hepatitis C Virus***	Negative
	***Aspergillus *** **IgM/IgG antibodies and ** ***Aspergillus*** ** galactomannan**		Negative
	***Candida *** **IgM/IgG antibodies and ** ***Candida*** ** manan**		Negative
	**parasites**	***Echinococcus *** **spp.**	Negative
		***Taenia solium***	Negative
		***Toxocara canis***	Negative
	**protozoa**	***Toxoplasma gondii***	Negative
**Stool examination for parasites and protozoa**	Negative on repeated sampling		
**Blood culture**	Negative on repeated sampling		
**QuantiFERON-TB Gold test**	Negative		

**Figure A1:**
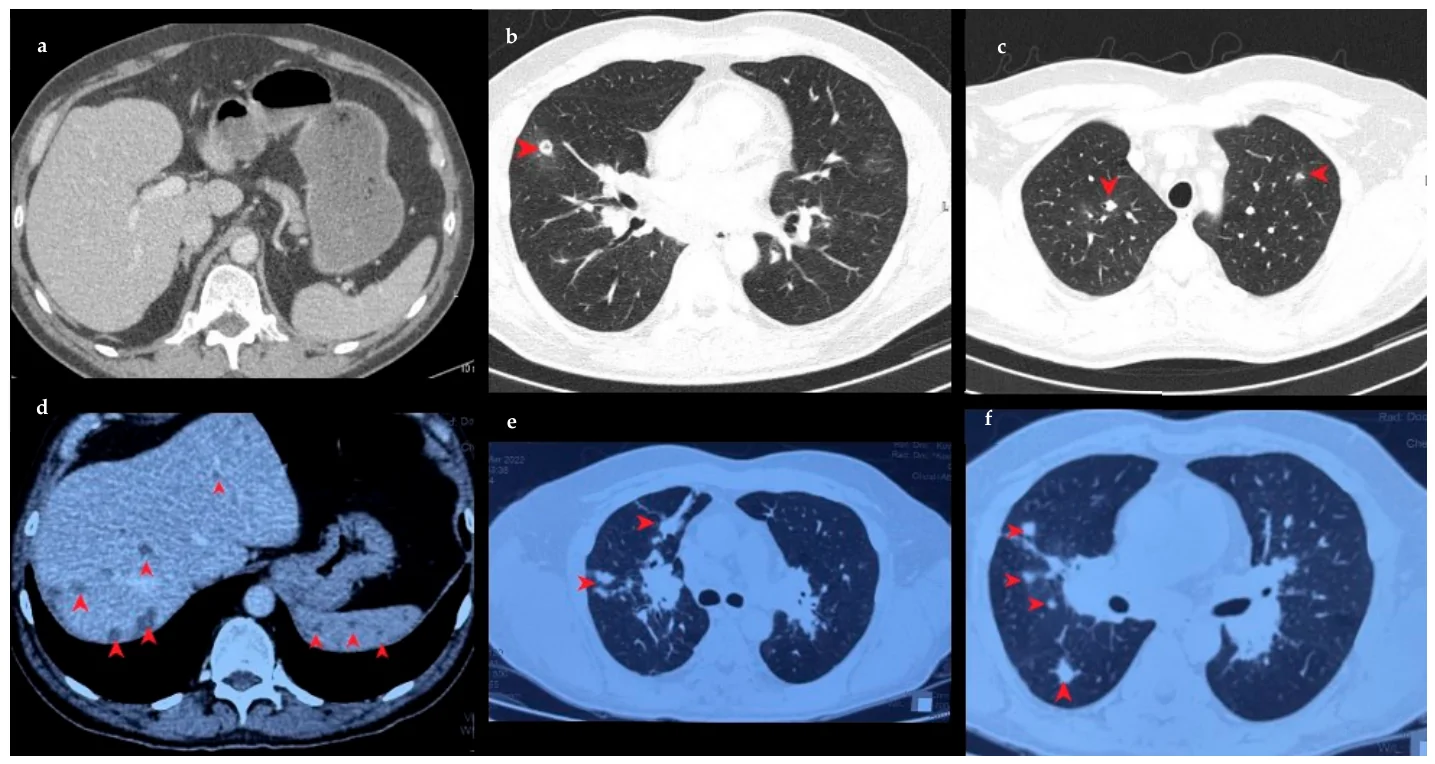
Radiological findings through time: (**a**) CT abdomen (2018) showing normal liver and spleen parenchyma without focal changes; (**d**) CT abdomen (2022) showing multiple hypodense clearly defined de novo focal changes in liver and spleen parenchyma (arrows); (**b**,**c**) chest CT (2018) showing mediastinal lymphadenopathy—single lymph nodes and conglomerates (the biggest being a subcarinal lymph node measuring at 59 × 23 mm), diffuse micronodular and nodular changes in lung parenchyma with signs of excavation (arrow on (**b**)) and surrounding ground-glass opacity; (**e**,**f**) chest CT (2022) showing mediastinal bilateral conglomerates of lymph nodes (largest node at 32 mm) and progression in size and number of nodular changes with excavation in lung parenchyma (arrows).

**Figure A2:**
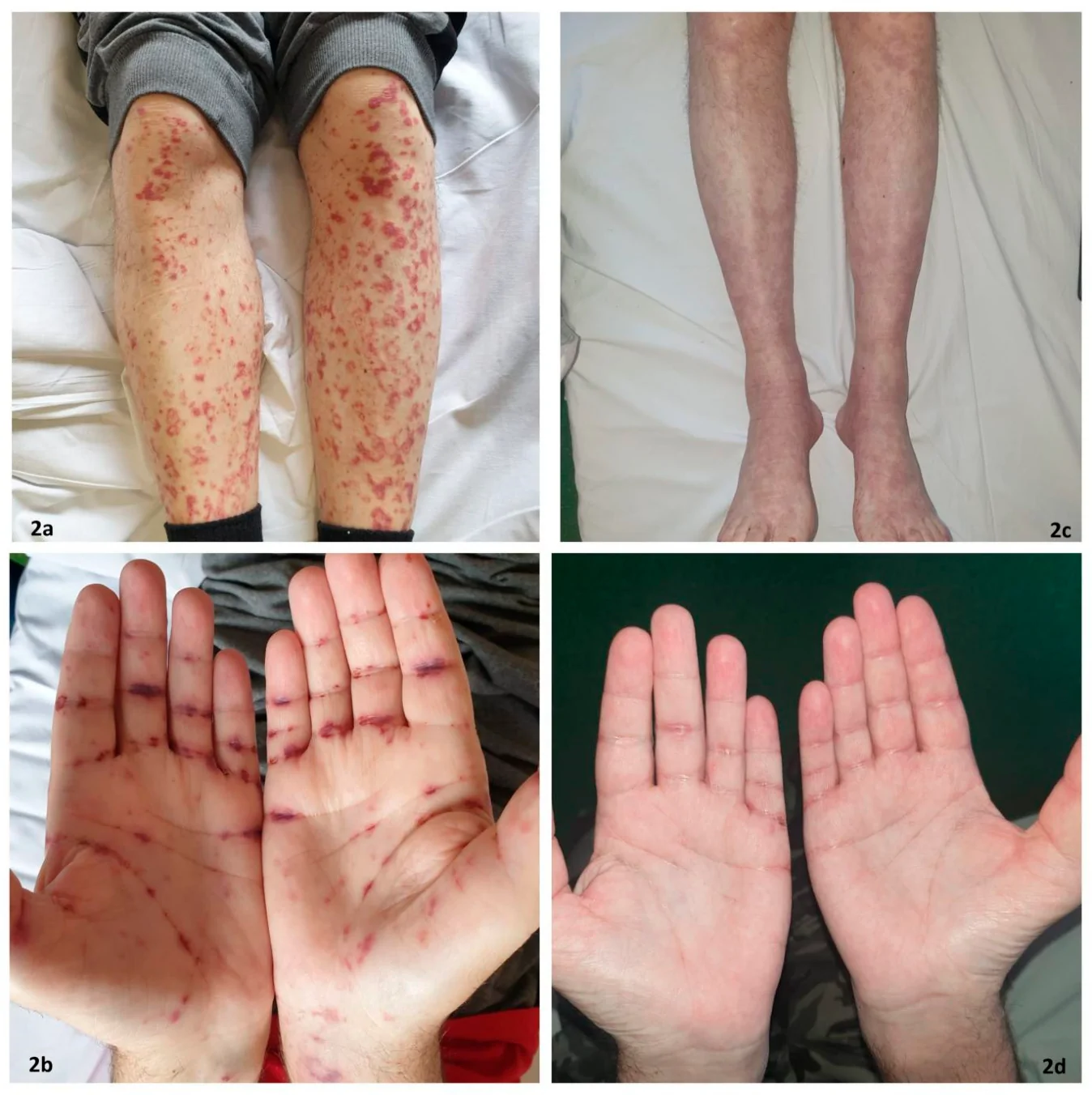
Cutaneous examination revealed purpuric lesions on the lower extremities (**a**) and hands (**b**), which markedly improved following treatment initiation (**c**,**d**).

**Figure A3:**
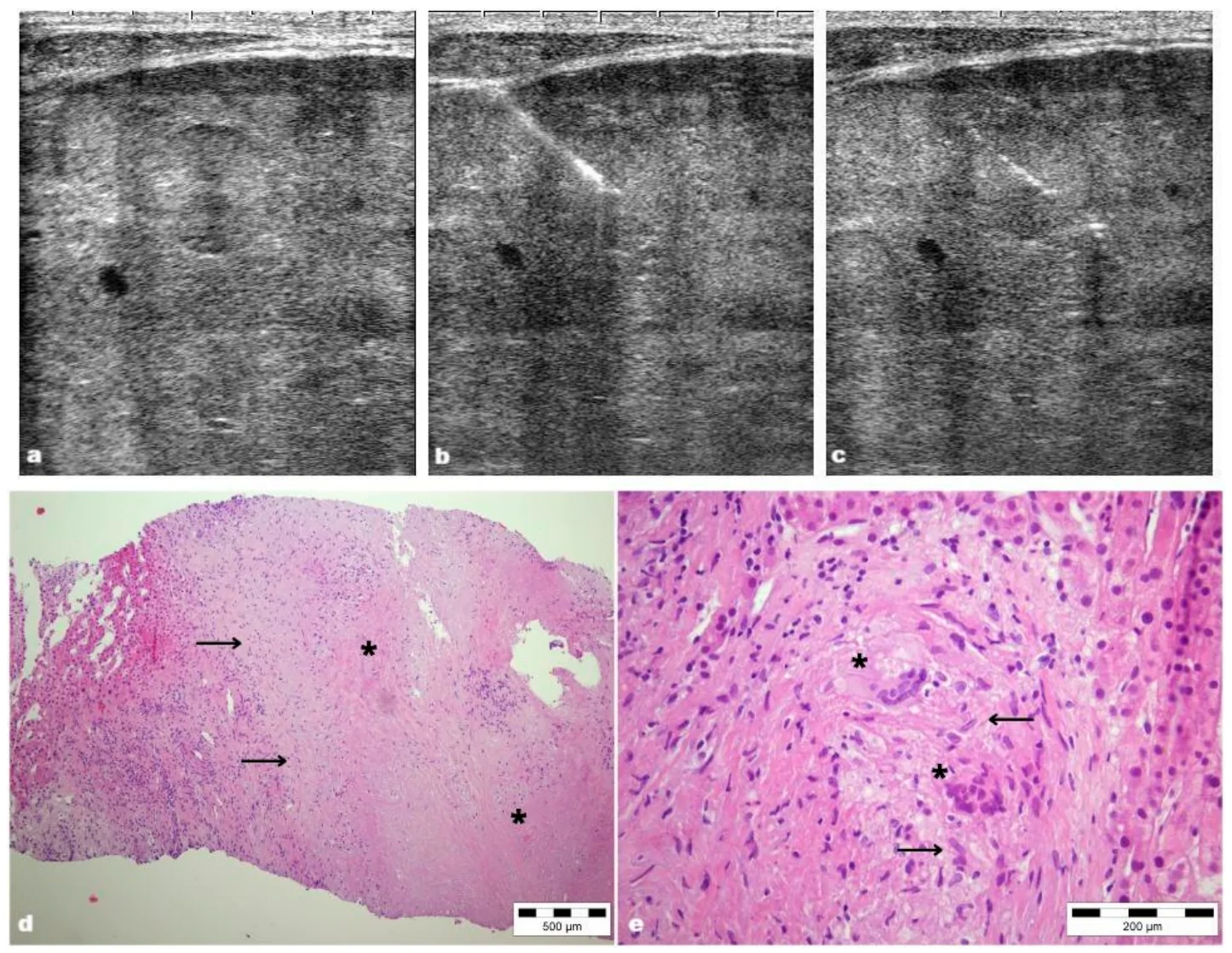
Panels in the top row (**a**–**c**) correspond to percutaneous core biopsy of liver: (**a**) ultrasound image showing an oval non-homogeneous lesion within the liver parenchyma (original magnification at 1:2); (**b**) positioning of the true-cut biopsy needle tip centrally within the targeted lesion under ultrasound guidance (original magnification at 1:2); (**c**) post-biopsy ultrasound demonstrating multiple gas inclusions at the biopsy site, a common and expected postprocedural finding (original magnification at 1:2); panels in the bottom row (**d**,**e**) show histopathological findings: (**d**) a larger necrotizing granuloma comprised mostly of epithelioid cells (arrows) with central necrosis and fibrosis (*). Surrounding liver tissue is seen in the upper left corner. Hematoxylin and eosin stain, original magnification ×100; (**e**) granuloma consisting of epithelioid cells (arrows) and multinucleated giant cells (*). Hematoxylin and eosin stain, original magnification ×400.

**Figure A4:**
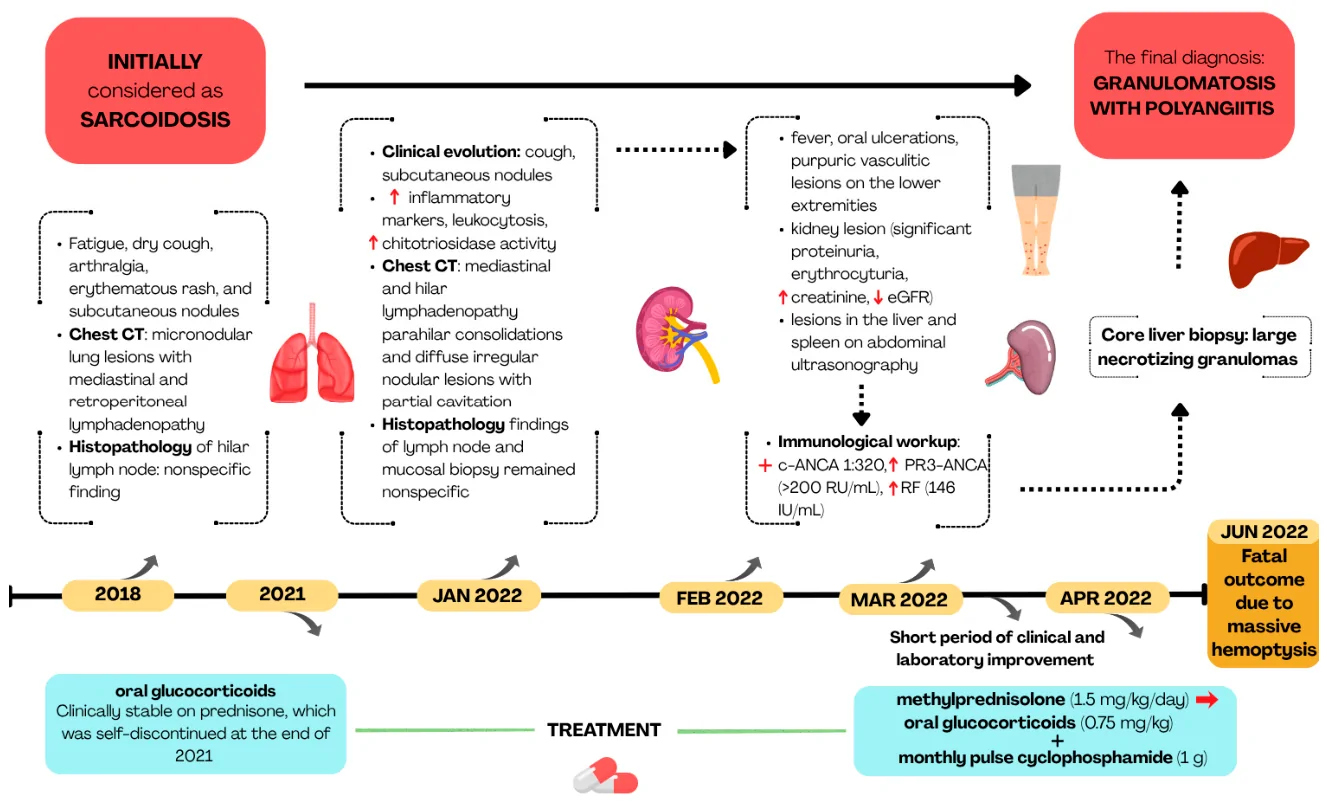
Timeline illustrating the patient’s clinical course.

## Data Availability

The data presented in this case report are not publicly available due to patient privacy and ethical restrictions. All relevant data supporting the findings of this case report are included within the article.

## Supplementary Materials

The following supporting information can be downloaded at https://www.mdpi.com/article/10.3390/diseases14090328/s1. File S1: CARE Checklist of information to include when writing a case report.
